# Internet-Delivered Cognitive Behavioral Therapy for Children With Pain-Related Functional Gastrointestinal Disorders: Feasibility Study

**DOI:** 10.2196/mental.7985

**Published:** 2017-08-10

**Authors:** Maria Lalouni, Brjánn Ljótsson, Marianne Bonnert, Erik Hedman-Lagerlöf, Jens Högström, Eva Serlachius, Ola Olén

**Affiliations:** ^1^ Department of Medicine, Solna Karolinska Institutet Stockholm Sweden; ^2^ Stockholm Health Care Services Stockholm County Council Stockholm Sweden; ^3^ Department of Clinical Neuroscience Division of Psychology Karolinska Institutet Stockholm Sweden; ^4^ Centre for Psychiatry Research Department of Clinical Neuroscience Karolinska Institutet Stockholm Sweden; ^5^ Department of Medical Epidemiology and Biostatistics Karolinska Institutet Stockholm Sweden; ^6^ Department of Clinical Neuroscience Osher Center for Integrative Medicine Karolinska Institutet Stockholm Sweden; ^7^ Department of pediatric gastroenterology and nutrition Sachs’ Children’s hospital Stockholm Sweden

**Keywords:** cognitive therapy, behavior therapy, functional gastrointestinal disorders, abdominal pain, irritable bowel syndrome

## Abstract

**Background:**

Pain-related functional gastrointestinal disorders (P-FGIDs; eg, irritable bowel syndrome) are highly prevalent in children and associated with low quality of life, anxiety, and school absence. Treatment options are scarce, and there is a need for effective and accessible treatments. Internet-delivered cognitive behavior therapy (Internet-CBT) based on exposure exercises is effective for adult and adolescent irritable bowel syndrome, but it has not been evaluated for younger children.

**Objective:**

The objective of this study was to assess acceptability, feasibility, and potential clinical efficacy of Internet-CBT for children with P-FGIDs.

**Methods:**

This was a feasibility study with a within-group design. We included 31 children aged 8-12 years and diagnosed with P-FGID, according to the ROME III criteria. Mean duration of abdominal symptoms at baseline was 3.8 years (standard deviation [SD] 2.6). The treatment was therapist-guided and consisted of 10 weekly modules of exposure-based Internet-CBT. The children were instructed to provoke abdominal symptoms in a graded manner and to engage in previously avoided activities. The parents were taught to decrease their attention to their children’s pain behaviors and to reinforce and support their work with the exposures. Assessments included treatment satisfaction, subjective treatment effect, gastrointestinal symptoms, quality of life, pain intensity, anxiety, depression, and school absence. Data were collected at pretreatment, posttreatment, and 6-month follow-up. Means, standard errors (SEs), and Cohen *d* effect sizes were estimated based on multi-level linear mixed models.

**Results:**

Most children 25/31 (81%) completed 9 or 10 of the 10 treatment modules. Almost all children, 28/31 (90%), reported that the treatment had helped them to deal more effectively with their symptoms, and 27/31 (87%) children declared that their symptoms had improved during the treatment. Assessments from the parents were in accordance with the children’s reports. No child or parent reported that the symptoms had worsened. We observed a large within-group effect size on the primary outcome measure, child-rated gastrointestinal symptoms from pretreatment to posttreatment (Cohen *d*=1.14, *P*<.001, 95% CI 0.69-1.61), and this effect size was maintained at 6-month follow-up (Cohen *d*=1.40, *P*<.001, 95% CI 1.04-1.81). We also observed significant improvements from pretreatment to posttreatment on a wide range of child- and parent-rated measures including quality of life, pain intensity, anxiety, depression, and school absence. All results remained stable or were further improved at 6-month follow-up.

**Conclusions:**

This study shows that children with longstanding P-FGIDs, and their parents, perceive exposure-based Internet-CBT as a helpful and feasible treatment. The included children improved significantly despite a long duration of abdominal symptoms before the intervention. The treatment shows potential to be highly effective for P-FGIDs. The results need to be confirmed in a randomized controlled trial (RCT).

## Introduction

Pain-related functional gastrointestinal disorders (P-FGIDs) according to the Rome III criteria are characterized by persistent or recurrent abdominal pain without an organic explanation. P-FGIDs include irritable bowel syndrome (IBS), functional abdominal pain (FAP), and functional dyspepsia (FD). IBS is most common and is by definition associated with fecal disturbances. In FAP, pain is often the only symptom, and in FD the pain is located in the upper abdomen and is often accompanied by symptoms like early satiety and nausea [1]. P-FGIDs affect about 13% of all children [2] and are associated with anxiety, depression [3], school absence, parental work absence, low quality of life [4], and extensive health care visits [5]. Support for the efficacy of medical [6] and dietary [7] treatments is weak, and there is a lack of treatment options in regular health care for these children. Cognitive behavior therapy (CBT) has been shown to be effective for P-FGIDs [7], but since CBT often includes multiple components, it is unclear which ones are effective [8]. A newly published Cochrane review concludes that identifying active components of psychological interventions in treatments for recurrent abdominal pain is an area of priority [9].

One potentially efficacious psychological intervention for pediatric P-FGIDs is exposure therapy. This treatment is based on a model proposing that P-FGID-related stimuli have been associated with pain, fear, or other unpleasant feelings such as losing control (ie, a respondent conditioning process [10,11]). The stimuli are typically avoided to reduce these experiences (ie, an operant conditioning process). Avoided stimuli can include abdominal symptoms, certain foods that are associated with abdominal symptoms, and situations in which abdominal symptoms are perceived as particularly intolerable. The avoidance prevents the child from gaining new and possibly contradictory experiences of the stimuli, which in turn contributes to maintenance of the fear of the stimuli and maintenance of the symptoms. This is consistent with what has been shown in adult studies: avoidance and control of symptoms seem to maintain the abdominal problems [12,13]. Exposure-based CBT includes exercises where the patient provokes the feared stimuli and approaches avoided situations in a graded manner. Examples of exposure exercises are eating symptom-provoking foods, postponing toilet visits, participating in previously avoided activities in the presence of symptoms, and decreasing medication for abdominal symptoms. Exposure therapy may be perceived to be difficult or aversive for children to engage in, and studies show that psychologists, even those using a behavioral approach, are often hesitant to include exposure in their treatments [14]. However, our previous study of exposure-based CBT in face-to-face format showed that children were adherent to the treatment and considered the exposure exercises to be helpful in dealing with symptoms [15]. Exposure-based CBT has also been proven effective for adults and adolescents with IBS [16-19] and shows promising results for children with P-FGIDs [15].

Parents are probably the most important contextual factor for younger children, and parents’ behavioral responses and coping mechanisms have been related to children’s pain symptoms [20]. In an experimental study, Walker et al [21] showed that parental attention to their child’s pain expressions increased both the child’s pain complaints and self-assessed abdominal symptoms after the experiment. It is therefore important to address parental behavior in a treatment for children with P-FGIDs, and this approach has been used in several treatment studies [22-24]. In exposure-based CBT, parents facilitate and encourage their child’s work with exposure exercises and reinforce and model adaptive behavior.

One major challenge in somatic health care is that the availability of CBT-trained psychologists is low [25]. Internet-delivered CBT (Internet-CBT) could be a viable option to make effective treatments more available to children. Internet-CBT has several advantages compared with therapy delivered in face-to-face format, such as being independent of geographical distance, requiring less therapist time, and being cost-effective [26]. Internet-CBT has also been shown to enable an as good working alliance between children and therapists as CBT delivered face-to-face [27]. Furthermore, the standardized format of Internet-CBT makes it possible to deliver the treatment with high treatment fidelity, and families are able to participate in the treatment without taking time off from school or work [8]. Exposure-based Internet-CBT has been shown to be effective for adult and adolescent IBS [16-19] and promising for adolescents with P-FGIDs [28]. It has also been proven effective for other disorders in children and adolescents like anxiety disorders [29] and obsessive compulsive disorder (OCD) [30]. However, to the best of our knowledge, there are no studies of exposure-based CBT delivered via Internet for children aged 8-12 years with P-FGIDs [8]. In this study, we therefore aimed to assess the feasibility, acceptability, and potential clinical efficacy for such a treatment in preparation for a forthcoming randomized controlled trial (RCT).

## Methods

### Design

This was a feasibility study with a pre- posttest design that included 31 children with P-FGIDs who were 8-12 years old. The study is reported according to the TREND statements for evaluations with nonrandomized designs. It was approved by the regional ethics review board in Stockholm, Sweden July 24, 2015 (2015/969-31) and registered at ClinicalTrials.gov June 17, 2015 (NCT02475096).

### Inclusion Criteria

The inclusion criteria were (1) age ≥8 and <13 years; (2) IBS, FAP, or FD diagnosis according to the Rome III criteria; (3) no more than 40% school-absenteeism; (4) stable dose since at least one month if treated with psychopharmacological medications; and (5) normal reading and writing skills (the child and the parent responsible for treatment and assessments). Exclusion criteria were (1) nonfunctional medical conditions that better explained the child’s abdominal symptoms (eg, celiac disease), (2) other ongoing psychological treatment, and (3) severe psychosocial or psychiatric problems that needed immediate attention. School-absenteeism of more than 40% (criteria [c]) was considered an acute and serious problem in need of more intensive care than an Internet-delivered intervention study can offer. Excluded children in need of other care were referred to other health care providers.

### Procedure

Participants were included in a nation-wide recruitment from August 2015 to January 2016. Follow-up assessments were collected from June to November 2016. The study was conducted at the Child and Adolescent Psychiatry Research Center in Stockholm. Physicians within primary, secondary, or tertiary care, who were informed about the study via emails and lectures, referred children to the study. Physicians signed a health form in which they confirmed the P-FGID diagnosis and reassured that basic work-up had been normal (normal linear growth and no involuntary weight stagnation or loss, negative tests for transglutaminase lgA antibodies, and in case of diarrhea for fecal calprotectin). The parents were contacted via telephone, and inclusion and exclusion criteria were assessed. The P-FGID phenotype was confirmed by a self-assessment version of the Rome III form that was completed by the families via the Internet and in a clinical interview at the research clinic conducted by the study’s psychologists. During the clinical interview, psychiatric disorders were assessed with the Mini-International Neuropsychiatric Interview for Children and Adolescents (MINI-KID) [31,32]. Written informed consent was obtained from the parents, and verbal informed consent was obtained from the child. During one part of the interview the child was asked questions without the parents present in the room. These questions concerned school, friends, family, and if the child had ever been mistreated. After the clinical interview, the child was either included or excluded. A child and adolescent psychiatrist (ES) and pediatric gastroenterologist (OO) were available for consultation if there were uncertainties regarding the child’s mental or physical health.

### Intervention

The therapist-guided Internet-CBT used in this study was based on the treatments for adults and adolescents developed by members of the research group [28,33]. It was adapted for children and tested in a face-to-face treatment study before this trial [15]. The treatment consisted of ten modules for the children and ten modules for the parents, delivered once a week. An overview of the treatment is presented in Table 1.

**Table 1:** Overview of the treatment.

Module	Child	Parent
1	Psycho-education about abdominal symptoms. Explanatory model of symptoms and treatment (Figure 1). Mapping avoidant and controlling behaviors. Setting goals.	The role of parental attention. Validating the child’s experience and shifting focus. Mapping parental behaviors. Handling worry and frustration.
2	The role of thoughts. A short mindfulness exercise, “SOL” (Stop, Observe, Let go). Building an exposure hierarchy.	“Golden moments”—spending quality time with the child without focusing on abdominal symptoms.
3	Functional analyses. Psycho-education about exposure. Exposure exercises.	Supporting the child in the treatment. Introduction of token game. Increasing school attendance.
4	Review of first exposure exercises. Toilet habits. Functional analyses. Exposure exercises.	How to handle parental stress. Plan for own recreational activities.
5	Review of the treatment sessions 1-4. Exposure exercises.	Review of the treatment sessions 1-4. Inventory of parental challenges.
6	Functional analyses of goal-directed behaviors. Exposure exercises, increasing the difficulty—level up.	Solving problems with the treatment together with the child.
7	Functional analyses of goal-directed behaviors. Review of the goals. Exposure exercises.	Functional analyses of parental behavior with emphasis on the interaction between parent and child. Functional analysis of goal-directed behaviors.
8	Positive analyses of goal-directed behaviors. Increasing the difficulty—exposure to multiple stimuli.	Review of treatment, part I. Rewarding yourself for the hard work with the treatment.
9	Quizzes of the treatment. Review of what has been accomplished so far.	Review of treatment, part II. Lessons learned. Review of parental challenges.
10	Review of avoidant and controlling behaviors, goals, and hierarchy. Maintenance and relapse prevention.	Review of parental behaviors. Maintenance and relapse prevention.

One parent was responsible for the treatment and was instructed to review the child’s modules together with the child and to share the content of the parental and child modules with the other parent. The parent responsible for the treatment also completed the parental self-assessments. All children and parent modules included homework exercises that were reviewed in the subsequent module. Case examples were used throughout the children’s modules, modeling the exercises, including behavior mapping, goal setting, and exposure exercises.

**Figure 1:**
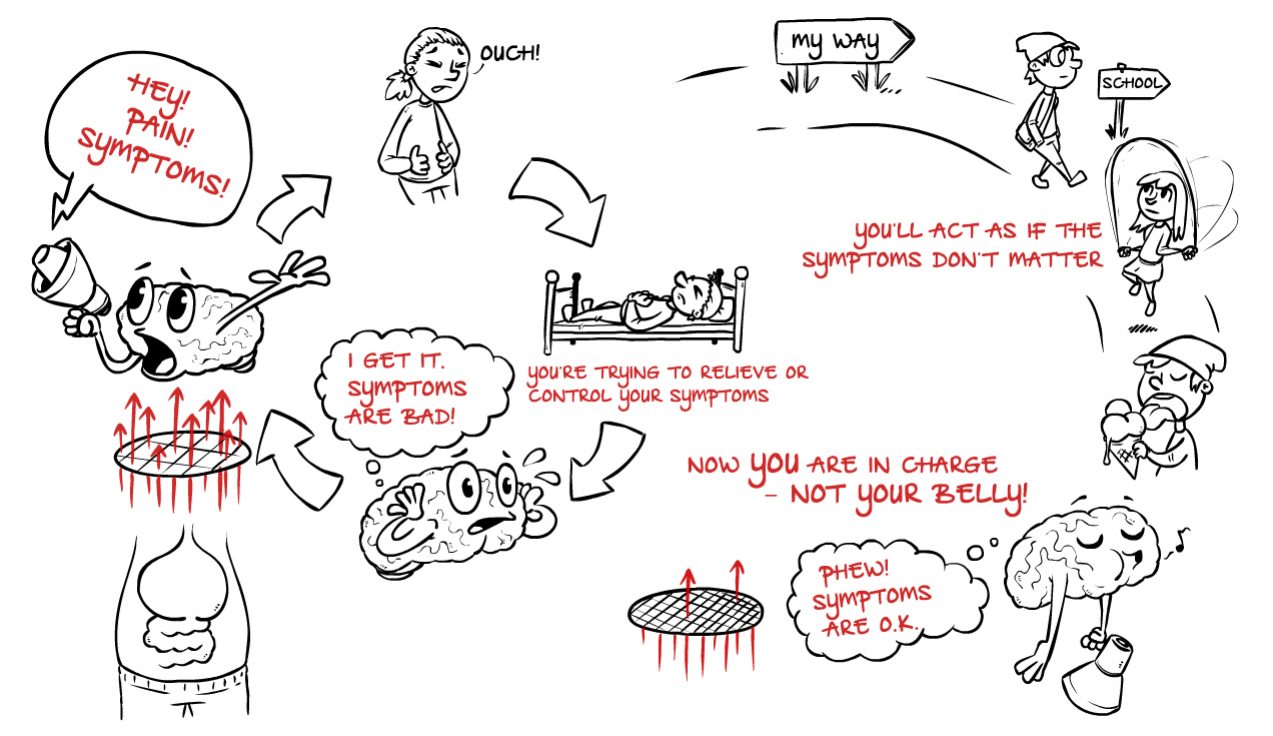
Explanatory model of abdominal pain and treatment presented to the child (translated from Swedish).

#### Children’s Modules

An explanatory model of the maintenance of abdominal symptoms and the exposure-based treatment approach was presented as an animated film (Figure 1).

The model uses a metaphor of a porous filter between the stomach and the brain to explain the children’s hypersensitivity toward abdominal signals. The brain is compared with a loudspeaker that amplifies the abdominal signals because they are perceived as important or even dangerous. The increased hypervigilance is explained as a consequence of the behavioral responses to control or avoid the symptoms, for example, resting, avoiding activities, or rushing into the bathroom. These behaviors confirm the importance or danger of the abdominal signals, leading the brain to become more attentive toward the signals: a vicious circle has been established. Exposure to abdominal symptoms is presented as a means to break the vicious circle. During the exposure exercises, the children provoke pain and other abdominal symptoms and engage in goal-directed behaviors with the long-term purpose of decreasing symptoms and regaining control. The children are told that one major purpose of the treatment is that they, and not their bellies, should be in charge.

**Figure 2:**
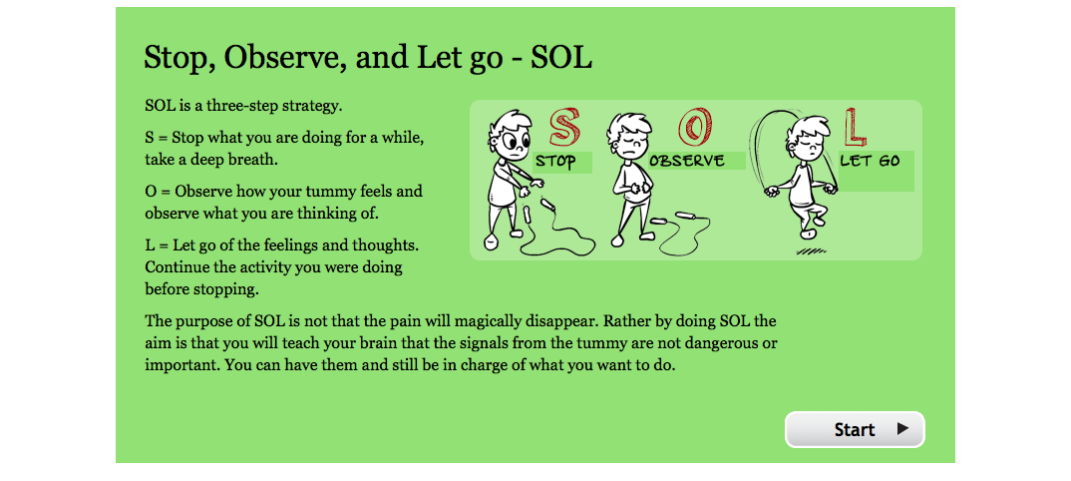
Screenshot from the treatment platform showing the mindfulness exercise “Stop, Observe, and Let go” (translated from Swedish).

**Figure 3:**
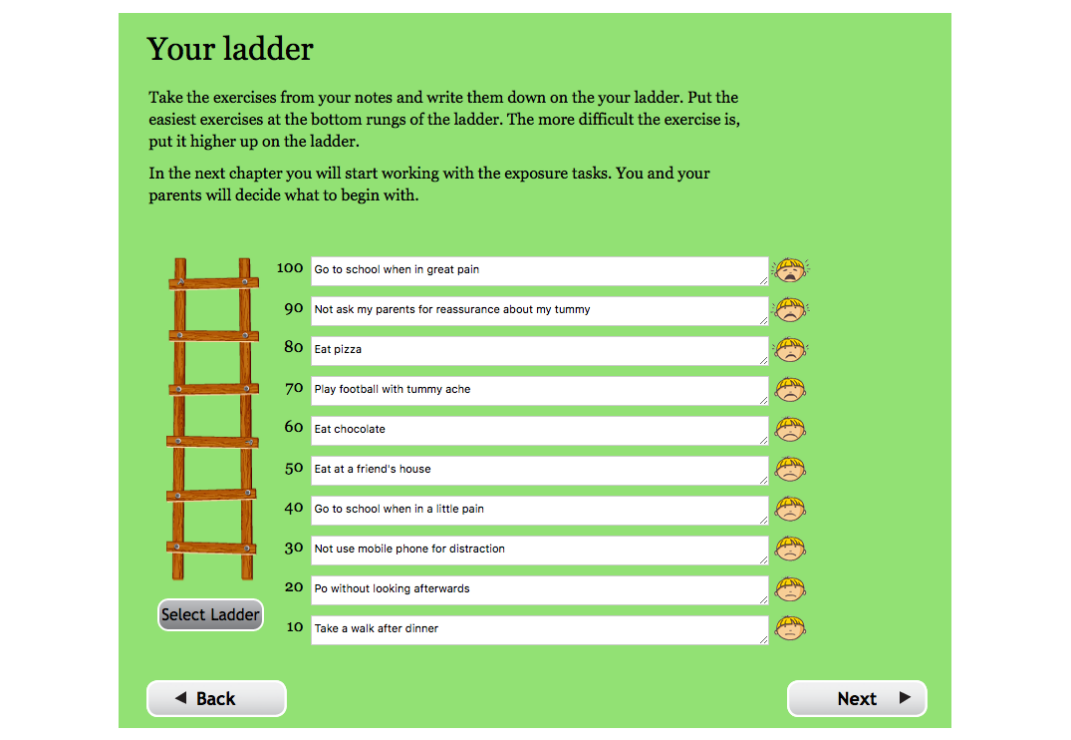
Screenshot from the treatment platform showing a hierarchy of exercises (translated from Swedish).

A short mindfulness exercise “SOL” was presented as a way to increase the effect of the exposures and to engage in goal-directed behaviors. Thoughts were presented as something that is difficult to control and that one way to handle catastrophic thoughts is to observe them and identify them as unhelpful and try not letting them interfere with ongoing behavior. The children were taught to (1) stop what they were doing, (2) observe their thoughts and symptoms for a short while, and (3) let go: continue to do whatever they were doing, in the presence of the thoughts and symptoms. SOL is presented in Figure 2.

The children mapped their avoidance and controlling behaviors. Exposure exercises that aimed to break these behaviors were planned and placed on a hierarchy; see Figure 3. The hierarchies were used to increase the difficulty of the exposures as they advanced through the treatment. Functional analyses of avoidance and controlling behaviors, as well as positive analyses for goal-directed behaviors were conducted throughout the treatment.

#### Parents’ Modules

**Figure 4:**
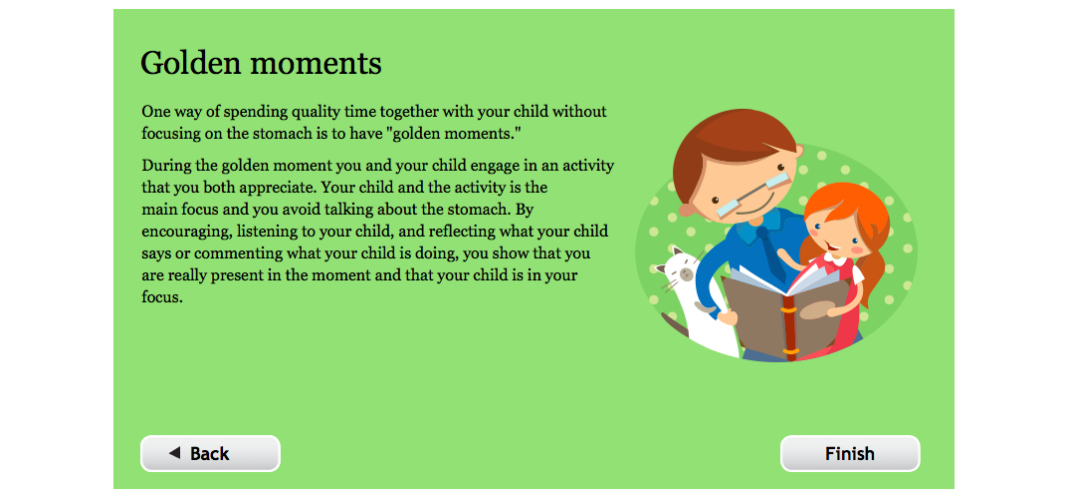
Screenshot from the treatment platform showing golden moments (translated from Swedish).

The parents received information on how attention and giving privileges may reinforce the child’s perception of pain and pain behavior. Common strategies used in parenting programs were introduced as a means to reinforce children’s adaptive behaviors [34,35]. The strategies included validating the child’s experience of abdominal symptoms and then shift focus to the activity; spending quality time together without focusing on the stomach, so called “golden moments,” see Figure 4; taking breaks if the parent was unable to act in a calm way when the child expressed symptoms; and using encouragement as well as a token game to reinforce the child’s work with exposures. The token game consisted of a printed game board where the child marked completed exposures with a pen and received small rewards for every fourth to eighth exposure on the way to the goal, where the child usually received a somewhat larger reward. The parents were given examples of rewards, such as letting the child choose what to have for dinner or to play a game together. The overall aim of the parental modules was to help the parents support the child with the exposure exercises and to reduce reinforcement of behaviors that are counter-productive to the exposure-based approach, such as avoidance and control of symptoms.

#### Therapists

Every family had an assigned clinical psychologist whom they had met during the initial clinical interview. New treatment modules were provided every Friday, and the participants were instructed to complete the modules during the weekend. On Mondays, the psychologists reviewed the work and provided written feedback within the platform. On the other weekdays, the psychologists reminded participants that had been inactive and had an ongoing communication with the participants via the platform. All therapists (ML, MB, and JH) were licensed psychologists with 8-9 years of experience of CBT and had 1-4 years of experience of Internet-CBT with children and adolescents. ML provided supervision on demand to the other psychologists throughout the study.

### Outcome Measures

The assessments were self-administered and provided via a secure platform over the Internet. The child and the parent responsible for the treatment made assessments at screening, pretreatment, posttreatment, and at 6-month follow-up. Some measures were assessed weekly during treatment by the children: Pediatric Quality of Life Inventory Gastrointestinal Symptom Scale (PedsQL Gastro), FACES Pain Rating Scale, pain-free days, and IBS-Behavioral Responses Questionnaire (IBS-BRQ), whereas parents assessed IBS-BRQ weekly; see descriptions of the measures below. The weekly assessments were included as a part of the piloting of a forthcoming randomized trial. Therefore, only a figure of the weekly assessments of the main outcome was included in this study (Figure 8). Parents were instructed to help their child during the assessment if they needed help, without influencing the child’s answers. The pediatric initiative on methods, measurement, and pain assessment in clinical trials (PedIMMPACT) recommendations for clinical trials for recurrent pain were used as guidelines in choosing measurements for the study [36].

#### Child-Rated Outcome Measures

PedsQL Gastro rated by the child was the primary outcome. It is a 9-item scale assessing last month’s gastrointestinal symptoms. The scale was developed to assess symptoms that are common in P-FGID disorders [37]. The 5-point scale ranges from never (0) to almost always (4). The items are reversely scored and transformed to a 0-100 scale, with higher scores indicating less symptoms.

Pediatric Quality of Life Inventory (PedsQL QOL) is a 23-item scale assessing quality of life for children aged 8-12 years, showing good validity and reliability [38]. The scoring is identical to PedsQL Gastro. Higher scores indicate greater quality of life.

Faces Pain Scale-Revised (FPS-R) was used to assess pain intensity. Human faces showing pain expressions corresponding to numbers from no pain (0) to worst pain (10) help the child rate last week’s worst pain intensity. The scale has been validated for children [39].

Pain-free days was assessed by asking about how many days last week the child had no pain or only so little pain that he or she felt okay [36].

Child Depression Inventory-Short version (CDI-S) assesses depressive symptoms in children. For each of the 10 items, there are three statements corresponding to 0, 1, or 2 points, with higher scores indicating more problems with depressive symptoms [40,41] *.*

Spence Children Anxiety Scale (SCAS) is a 45-item scale that assesses anxiety in children aged 8-12 years [42]. In this study, a hitherto unpublished short version with 18 items was used (SCAS-S). The frequency of anxiety symptoms is rated on a 4-point scale, with answers ranging from never (0) to always (3).

Visceral sensitivity Index (VSI *)* assesses gastrointestinal specific anxiety and was a process variable in this study [12]. It was developed for adults with IBS, and some wordings were changed to fit the pediatric P-FGID population. It comprises 15 items and is rated on a 6-point scale ranging from strongly disagree (0) to strongly agree (5).

IBS-BRQ is validated for adults with IBS and has shown high internal consistency for that group (Cronbach alpha=.86) [43]. In this study, a child-adjusted version of the scale with 11 items was used. The scale assesses avoidant behavior and controlling of symptoms and was a process variable in this study. The items are rated on a 7-point scale with only endpoints defined: never (1) and always (7).

The catastrophizing subscale of the Pain Response Inventory was used to assess maladaptive coping by catastrophizing. It consists of 5 items rated in 5 points ranging from never (0) to always (4) [44].

Children’s Somatization Inventory-24 (CSI-24 *)* is a 24-item scale that assesses perceived severity of somatic symptoms. Items include symptoms such as headache, sore muscles, and gastrointestinal symptoms. It is a 5-point scale with responses ranging from not at all (0) to a whole lot (4). CSI-24 has been evaluated for a pediatric population and was found to be psychometrically sound [45]. Seven of the items assess gastrointestinal symptoms and were reported as a separate subscale, CSI-24 (gastro), as has been done in other studies [22,46].

Insomnia Severity Index-Child version (ISI-C) was used to assess problems with sleep. It comprises seven items covering different aspects of sleep problems and is rated on a 5-point scale from no problems or not at all (0) to very large problems or very much (4) [47].

Pressure Activation Stress Scale (PAS) assesses stress in children. It comprises 11 items rated on a 5-point scale ranging from never (0), to always (4) [48].

School absence was assessed with the question: “How many hours last month were you absent from school due to pain?” with the responses on a 4-point scale: 0 hours (0), 1-5 hours (1), 6-10 hours (2), and more than 10 hours (3)[15].

Client Satisfaction Questionnaire-8 (CSQ-8) was used to measure different aspects of treatment satisfaction. It is an 8-item scale where questions are rated from 1-4, corresponding to different answers for the questions [49].

Subjective Assessment Questionnaire (SAQ) assesses the participant’s subjective perception of the treatment effect by asking one question about how severe the symptoms are after treatment compared with before treatment. It is a 7-point scale ranging from very much better (6) to very much worse (0) [50].

#### Parent-Rated Outcome Measures

Parents completed parental versions of PedsQL Gastro, PedsQL QOL, FACES Pain Rating Scale, pain-free days, CSI-24, school absence, SAQ, and CSQ-8 described above and the following measures.

Parental work absence was assessed with the question: “How many days in the last month have you or another adult been home from work due to your child’s abdominal problems?” The responses were rated on a 4-point scale: 0 days (0), 1-5 days (1), 6-10 days (2), and more than 10 days (3).

Adult responses to children’s symptoms (ARCS) is a 29-item scale with 4 points (1-4) [51]. Endpoints are defined as never (1) and always (4). Parents are asked how often they respond with certain behaviors when their child has abdominal pain. ARCS is analyzed in subscales and was a process variable in this study. We used the subscales Protect and Monitor (age-adjusted versions), which have been shown to be sensitive to change [52,53].

Patient health questionnaire-9 (PHQ-9) assesses the parent’s own depressive symptoms in 9 items rated on a 4-point scale ranging from not at all (0) to almost every day (3) [54].

Generalized anxiety disorder assessment-7 (GAD-7) is a 7-item scale that assesses the parents’ symptoms of anxiety [55]. Like PHQ-9, the scale ranges from not at all (0) to almost every day (3).

Adverse events (AE) assess negative effects associated with the treatment. Each negative effect was described in free-form text and its severity from no negative effect (0) to very negative effect (3) was rated on two scales, how much the event affected the child at the time of its occurrence, and how much it affected the child at the time of the assessment (ie, to what extent the effect lingered) [18].

### Data Analyses

All analyses were performed in R (R Foundation for Statistical Computing), except for the McNemar test that was performed in Stata 13 (StataCorp LP). Pretreatment, posttreatment, and six-month follow-up data were included in piecewise linear mixed models analysis using all available data, that is, analyses were based on intent-to-treat. Separate slopes were estimated for the pre- to posttreatment assessment (Slope 1) and posttreatment to six-month follow-up assessment (Slope 2). Slopes 1 and 2 were then summed to form the estimated overall pre to six-month follow-up improvement. Cohen *d* within-group effect sizes were calculated by dividing the estimated change scores with the model-implied standard deviation. Effect sizes were categorized as suggested by Cohen [56]: *d*=0.2 represents a small effect size, *d*=0.5 a medium effect size, and *d*=0.8 a large effect size. CIs and *P* values for the effect sizes were obtained using bootstrap with 5000 replications. An improvement of ≥30% on the primary outcome measure was used to define clinically significant change, which is consistent with recommendations and cut offs used in other studies [57,58].

## Results

### Participants

**Figure 5:**
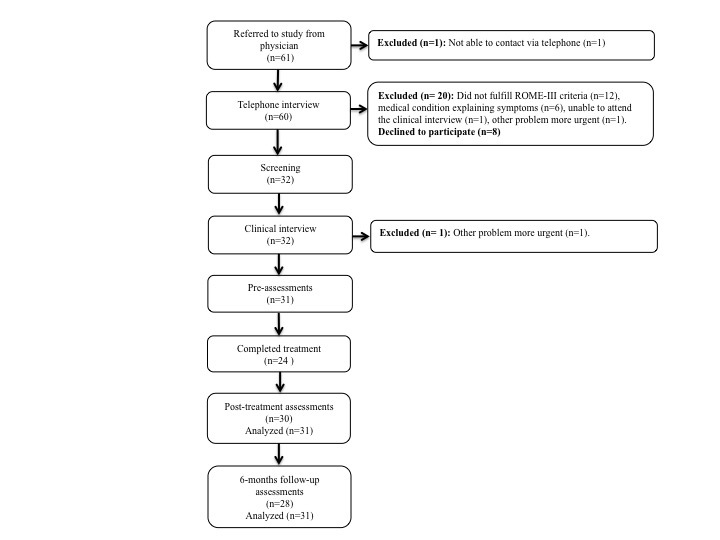
Participants flow through the study.

There were 61 children referred to the study of which 30 were excluded or declined to participate; see the participants flow through the study (Figure 5). Of the 31 children included in the study, 19 were girls. The mean duration of abdominal symptoms was 3.8 years (range 0.3-11.0). Complete baseline characteristics are presented in Table 2.

**Table 2:** Patient characteristics at baseline (N=31).

Characteristics		Mean	n (%)	Range
Age (years)		10.7		8-12
Duration abdominal problems (years)		3.8		0.3-11
Girls			19 (61)	
Born in Sweden			30 (97)	
Parental heredity^a^			10 (32)	
Medication for abdominal symptoms^b^			12 (39)	
School absence last month^c^			25 (81)	
Distance from home to clinic (kilometers)		172		5-907
**Rome III diagnosis**				
	Irritable bowel syndrome		18 (58)	
	Functional abdominal pain		11 (35)	
	Functional dyspepsia		2 (6)	
**Psychiatric comorbidity (MINI-KID^d^)**				
	Any psychiatric comorbidity		10 (32)	
	Anxiety disorder		7 (23)	
	Depression		2 (6)	
	Suicidal thoughts (all low level)		2 (6)	
	Attention deficit disorder		1 (3)	
**Referring care unit**				
	Primary care		2 (6)	
	Secondary care		19 (61)	
	Tertiary care		10 (32)	
**Education, parents**				
	High School <3 years		2 (6)	
	High School ≥3 years		8 (26)	
	College		20 (64)	
	Other		1 (3)	

^a^At least one parent with abdominal problems.

^b^Polyethylene glycol, lactitol monohydrate, simeticone, sterculia, and calcium carbonate or magnesium hydroxide.

^c^Due to abdominal pain.

^d^MINI-KID: Mini-International Neuropsychiatric Interview for Children and Adolescents.

### Attendance and Attrition

**Figure 6:**
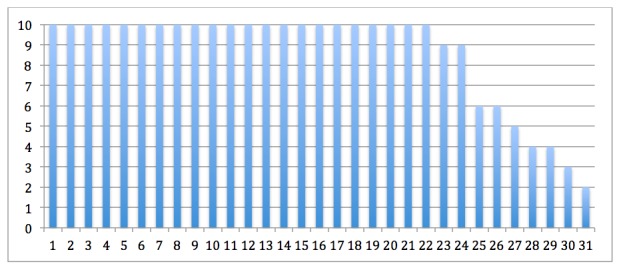
Number of modules completed by each of the 31 children. Dyads of children and parents share the same numbers on the x-axis in Figure 6 and 7.

**Figure 7:**
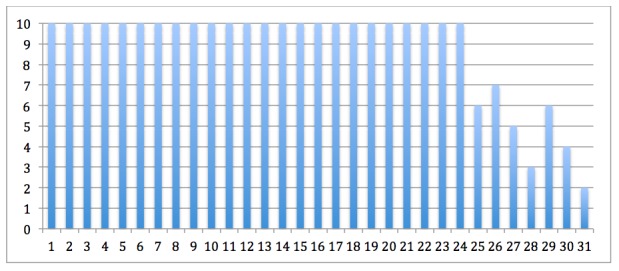
Number of modules completed by each of the 31 parents. Dyads of children and parents share the same numbers on the x-axis in Figure 6 and 7.

The mean number of modules that the children and their parents took part of was 8.6 and 8.8 of 10 modules, respectively. Most children and their parents completed 9-10 modules, 24/31 (77%), and were considered treatment completers. The noncompleters (n=7) were dyads where both children and parents completed 2-7 modules. Modules completed are illustrated in Figures 6 and 7. At the posttreatment assessments, there was almost no data attrition, 1/31 (3%). The one participant who did not respond to the posttreatment assessment was a noncompleter. At 6-month follow-up the data attrition was 3/31 (10%). Two of the participants who did not provide follow-up data were noncompleters, and one was a completer. Mean therapist time for the whole treatment was 165 (standard deviation [SD] 64.0) min per family, representing a mean of 19 min per week in treatment (mean therapist time divided by mean number of modules completed).

**Figure 8:**
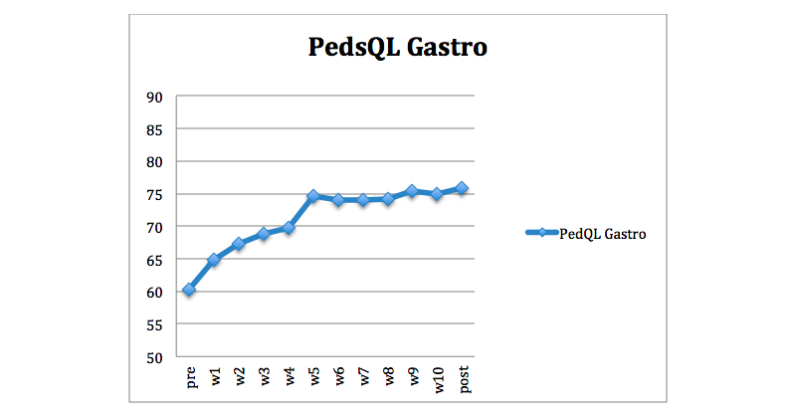
Observed means of child-rated gastrointestinal symptoms measured by PedsQL Gastro at pretreatment, every week during treatment, and at posttreatment. The scale ranges from 0-100, and the range in the sample was 25-100.

### Treatment Satisfaction and Subjective Treatment Effect

The children reported an average total score on the CSQ (range 8-32) of 25.1 (SD 5.1) and the parents an average total score of 28.1 (SD 4.4). Most children were satisfied with the support from the psychologist (28/31, 90%) and reported that the treatment had helped them deal more effectively with their symptoms (27/31, 87%). The children rated their mean subjective treatment effect on the SAQ (range 0-6) as 4.7 (SD 1.0), and the parents rated their child’s mean subjective treatment effect as 5.0 (SD 1.0). Of the 30 children who completed the postassessments, 26 reported that their symptoms had improved, and 4 reported that they were about the same as before treatment. These assessments were similar for the parents where 27 parents rated that their child’s symptoms had improved, and three rated that the symptoms were about the same as before treatment. No child or parent reported that the symptoms had worsened.

### Child-Rated Outcomes

Estimated means and SEs for all measures are presented in Table 3. Effect-sizes and their 95% CIs and *P* values are presented in Table 4. There was a significant pre- to posttreatment change on the primary outcome measure child-rated gastrointestinal symptoms (PedsQL Gastro). This change was maintained at 6-month follow-up. The within-group effect size was large *d*=1.14 (*P*<.001) from pre- to posttreatment and also from pretreatment to follow-up, *d*=1.40 (*P*<.001). At both posttreatment and at 6-month follow-up, 15/31 (48%) children reached clinically significant change on the primary outcome measure, defined as 30% improvement. The children who reached clinically significant change at posttreatment had a mean change score of 28.1 (SD=7.0, range=16.7-41.7) from pretreatment to posttreatment. For the children who reached clinically significant change at 6-month follow-up, the mean change score from pretreatment was 28.7 (SD=7.3, range=19.4-41.7). PedsQL Gastro was also assessed every week during treatment (Figure 8). All measures showed statistically significant improvement between pre- and posttreatment, except ISI-C (sleep problems) and PAS (stress) that showed improvement between pretreatment and 6-month follow-up. We also observed large effect sizes between pretreatment and posttreatment for quality of life (PedsQL QOL), gastrointestinal symptoms (CSI-24 [gastro]), and the process variables gastrointestinal specific anxiety (VSI) and avoidant behavior (IBS-BRQ). At 6-month follow-up, these effect sizes remained large and the effect sizes for pain intensity (FACES Pain Rating Scale), pain-free days, catastrophizing, and school absence had also become large compared with pretreatment. We observed a significant improvement of the effect sizes between posttreatment and 6-month follow-up for pain intensity (FACES Pain Rating Scale) *d*=0.56 (*P*=.01), catastrophizing *d*=0.69 (*P*<.001), gastrointestinal symptoms (CSI-24 [gastro]) *d*=0.40 (*P*=.03), and stress (PAS) *d*=0.57 (*P*=.006).

**Table 3:** Estimated means and standard errors at pretreatment, posttreatment, and 6-month follow-up reported by children.

Outcome measure	Pretreatment		Posttreatment		6-month follow-up	
	Mean	(SE^a^)	Mean	(SE)	Mean	(SE)
PedsQL Gastro^b,c^	60.30	(2.41)	75.63	(2.44)	79.08	(2.49)
PedsQL QOL^c,d^	72.48	(1.89)	85.75	(1.92)	87.56	(1.97)
FACES Pain Rating Scale	6.87	(0.43)	5.09	(0.44)	3.74	(0.45)
Pain-free days/week^c^	2.45	(0.36)	3.84	(0.36)	4.35	(0.38)
CDI-S^e^	2.90	(0.41)	1.92	(0.42)	1.86	(0.42)
SCAS-S^f^	12.45	(1.34)	10.27	(1.35)	9.13	(1.38)
VSI^g^	10.74	(1.06)	5.33	(1.07)	3.45	(1.10)
IBS-BRQ^h^	29.87	(1.67)	18.91	(1.69)	17.96	(1.74)
Catastrophizing	6.81	(0.67)	4.61	(0.67)	2.04	(0.69)
CSI-24^i^	15.48	(1.64)	11.78	(1.65)	9.17	(1.67)
CSI-24 (gastro)^j^	7.74	(0.62)	4.88	(0.63)	3.50	(0.64)
ISI-C^k^	6.03	(0.83)	5.19	(0.84)	3.97	(0.86)
PAS^l^	11.65	(1.20)	10.28	(1.22)	6.48	(1.25)
School absence^m^	1.45	(0.18)	0.81	(0.19)	0.59	(0.19)

^a^SE: standard error.

^b^PedsQL Gastro: Pediatric Quality of Life Inventory Gastrointestinal Symptom Scale.

^c^PedsQL Gastro, PedsQL QOL, and pain-free days are reversely scored. Higher scores indicate improvement.

^d^PedsQL QOL: Pediatric Quality of Life Inventory.

^e^CDI-S: Child Depression Inventory-Short version.

^f^SCAS-S: Spence Children Anxiety Scale-Short version.

^g^VSI: Visceral Sensitivity Index.

^h^IBS-BRQ: Irritable Bowel Syndrome-Behavioral Responses Questionnaire.

^i^CSI-24: Children’s Somatization Inventory.

^j^CSI-24 (gastro): Children’s Somatization Inventory-24 (gastro).

^k^ISI-C: Insomnia Severity Index-Child version.

^l^PAS: Pressure Activation Stress Scale.

^m^School Absence was rated in intervals of hours absent from school last month. 1=1-5 hours, 2=6-10 hours, and 3=more than 10 hours.

**Table 4:** Effects sizes for child-reported outcomes.

Outcome measure	Pre-post				Pre-FU6		
	Cohen *d*	(95% CI)	*P* value	Cohen *d*		(95% CI)	*P* value
PedsQL Gastro^a^	1.14^b^	0.69-1.61	<.001	1.40^b^		1.04-1.81	<.001
PedsQL QOL^c^	1.26^b^	0.82-1.72	<.001	1.43^b^		0.95-1.97	<.001
FACES Pain Rating Scale	0.74^b^	0.34-1.17	<.001	1.30^b^		0.81-1.74	<.001
Pain-free days	0.70^b^	0.25-1.17	=.002	0.95^b^		0.42-1.49	<.001
CDI-S^d^	0.43^b^	0.08-0.79	=.006	0.45^b^		0.06-0.87	=.005
SCAS-S^e^	0.29^b^	0.03-0.59	=.04	0.44^b^		0.06-0.87	=.002
VSI^f^	0.92^b^	0.56-1.31	<.001	1.24^b^		0.80-1.72	<.001
IBS-BRQ^g^	1.18^b^	0.76-1.65	<.001	1.28^b^		0.84-1.78	<.001
Catastrophizing	0.59^b^	0.17-1.00	=.002	1.29^b^		0.88-1.73	<.001
CSI-24^h^	0.41^b^	0.05-0.74	=.005	0.69^b^		0.43-0.97	<.001
CSI-24 (gastro)^i^	0.82^b^	0.49-1.17	<.001	1.22^b^		0.91-1.52	<.001
ISI-C^j^	0.18	−0.20 to 0.53	=.31	0.44^b^		0.11-0.69	=.01
PAS^k^	0.20	−0.16 to 0.60	=.31	0.77^b^		0.38-1.22	<.001
School absence	0.62^b^	0.26-1.05	<.001	0.84^b^		0.49-1.29	<.001

^a^PedsQL Gastro: Pediatric Quality of Life Inventory Gastrointestinal Symptom Scale.

^b^Significant effect sizes.

^c^PedsQL QOL: Pediatric Quality of Life Inventory.

^d^CDI-S: Child Depression Inventory-Short version.

^e^SCAS-S: Spence Children Anxiety Scale-Short version.

^f^VSI: Visceral Sensitivity Index.

^g^IBS-BRQ: Irritable Bowel Syndrome-Behavioral Responses Questionnaire.

^h^CSI-24: Children’s Somatization Inventory.

^i^CSI-24 (gastro): Children’s Somatization Inventory (gastro).

^j^ISI-C: Insomnia Severity Index-Child version.

^k^PAS: Pressure Activation Stress Scale.

#### School Absence

Before treatment, 25 of the 31 children (81%) reported that they had had some absence from school in the previous month related to abdominal symptoms. At posttreatment, 14 children (45%, 14/31) reported absence from school related to abdominal symptoms, and at 6-month follow-up, only 10/31 (32%) children reported absence from school in the previous month due to abdominal symptoms. McNemar tests showed that the differences in school absenteeism between pretreatment and posttreatment, and pretreatment and 6-month follow-up, were significant; *P*=.002 and *P*<.001, respectively. Of the 25 children who reported school absence at pretreatment, 10 children reported that they had no school absence at posttreatment, and 11 children reported that they had no school absence at 6-month follow-up. All children who reported no school absence at pretreatment also did so during the later assessments.

#### Rome III Criteria

At posttreatment, 6/31 (19%) children did not fulfill Rome III criteria according to their self-assessments any longer, and at 6-month follow-up, 16/31 (52%) no longer fulfilled the criteria. The distribution of the Rome III diagnosis at the different time points is presented in Table 5.

**Table 5:** Patients fulfilling Rome III criteria for different pain-related functional gastrointestinal disorders (P-FGID) diagnoses at pre, post, and 6-month follow-up.

Disorder	Pretreatment	Posttreatment	FU6
IBS^a^	19	4	2
FAP^b,c^	11	17	8
FD^c,d^	2	3	2
No P-FGID^e^	0	6	16

^a^IBS: irritable bowel syndrome.

^b^FAP: functional abdominal pain.

^c^Participants migrated between diagnoses, which explains the increase in functional abdominal pain (FAP) and functional dyspepsia (FD) between pre- and posttreatment.

^d^FD: functional dyspepsia.

^e^P-FGID: pain-related functional gastrointestinal disorders.

### Parent-Rated Outcomes

All parent-rated outcomes showed statistically significant improvements from pre- to posttreatment, except PHQ-9 (parental depression). Estimated means and SEs for all measures reported by parents are presented in Table 6. Effect-sizes and their 95% CIs and *P* values are presented in Table 7.

**Table 6:** Estimated means and standard errors at pretreatment, posttreatment, and 6-month follow-up reported by parents.

Outcome measure	Pretreatment		Posttreatment		6-month follow-up	
	Mean	(SE^a^)	Mean	(SE)	Mean	(SE)
PedsQL Gastro^b,c^	57.62	(2.22)	74.54	(2.25)	77.46	(2.29)
PedsQL QOL^c,d^	69.57	(2.05)	82.79	(2.07)	85.95	(2.12)
FACES Pain Rating Scale	6.19	(0.44)	3.91	(0.45)	3.03	(0.46)
Pain-free days/week^c^	2.32	(0.37)	3.71	(0.38)	5.20	(0.39)
CSI-24^e^	13.97	(1.08)	8.46	(1.09)	6.95	(1.11)
CSI-24 (gastro)^f^	8.55	(0.58)	5.29	(0.58)	3.55	(0.60)
School absence^g^	1.58	(0.19)	1.01	(0.19)	0.55	(0.19)
Work absence^h^	0.65	(0.10)	0.34	(0.10)	0.05	(0.10)
ARCS^i^protect	11.35	(0.88)	5.16	(0.90)	4.41	(0.92)
ARCS monitor	10.10	(0.57)	4.82	(0.58)	3.99	(0.59)
PHQ-9^j^	4.29	(0.74)	3.45	(0.74)	2.40	(0.75)
GAD-7^k^	3.26	(0.49)	1.90	(0.50)	1.83	(0.51)

^a^SE: standard error.

^b^PedsQL Gastro: Pediatric Quality of Life Inventory Gastrointestinal Symptom Scale.

^c^PedsQL Gastro, PedsQL QOL, and pain-free days are reversely scored. Higher scores indicate improvement.

^d^PedsQL QOL: Pediatric Quality of Life Inventory.

^e^CSI-24: Children’s Somatization Inventory-24.

^f^CSI-24 (gastro): Children’s Somatization Inventory (gastro).

^g^School Absence was rated in intervals of hours absent from school last month. 0=0 hours, 1=1-5 hours, 2=6-10 hours, and 3=more than 10 hours.

^h^Work Absence was rated in intervals of days home from work last month due to the child’s abdominal problems. 0=0 days, 1=1-5 days, 2=6-10 days, and 2=more than 10 days.

^i^ARCS: Adult Responses to Children’s Symptoms.

^j^PHQ-9: Patient Health Questionnaire-9.

^k^GAD-7: Generalized Anxiety Disorder-7.

Effect sizes were large from pretreatment to posttreatment for gastrointestinal symptoms (PedsQL Gastro), pain intensity (FACES Pain Rating Scale), quality of life (PedsQL QOL), somatization (CSI-24), and the process variables assessing parental responses to their children’s symptoms (ARCS protect and monitor). At 6-month follow-up, all measures showed significant improvements compared with pretreatment. All effect sizes were large from pretreatment to 6-month follow-up, except for parental depression (PHQ-9) and parental anxiety (GAD-7). We observed a significant improvement of the effect sizes between posttreatment and 6-month follow-up for pain-free days *d*=0.72 (*P*<.001), gastrointestinal symptoms (CSI-24 gastro) *d*=0.54 (*P*=.004), school absence *d*=0.44 (*P*=.006), and work absence *d*=0.52 (*P*=.02).

**Table 7:** Effects sizes for parent-reported outcomes; Cohen *d*, (95% CI), and *P* values.

Outcome measure	Pre-post			Pre-FU6		
	Cohen *d*	(95% CI)	*P* value	Cohen *d*	(95% CI)	*P* value
PedsQL Gastro^a^	1.37^b^	0.83-1.96	<.001	1.60^b^	1.03-2.22	<.001
PedsQL QOL^c^	1.16^b^	0.70-1.69	<.001	1.44^b^	0.93-1.97	<.001
FACES Pain Rating Scale	0.93^b^	0.46-1.42	<.001	1.29^b^	0.81-1.72	<.001
Pain-free days	0.67^b^	0.27-1.08	<.001	1.38^b^	0.90-1.88	<.001
CSI-24^d^	0.92^b^	0.44-1.38	<.001	1.17^b^	0.46-1.72	<.001
CSI-24 (gastro)^e^	1.02^b^	0.56-1.44	<.001	1.56^b^	1.18-1.96	<.001
School absence	0.55^b^	0.22-0.97	<.001	0.99^b^	0.60-1.49	<.001
Work absence	0.55^b^	0.00-1.11	=.01	1.07^b^	0.68-1.54	<.001
ARCS^f^protect	1.26^b^	0.67-1.79	<.001	1.41^b^	0.74-2.00	<.001
ARCS monitor	1.65^b^	0.96-2.35	<.001	1.91^b^	1.15-2.68	<.001
PHQ-9^g^	0.21	−0.13 to 0.50	=.16	0.46^b^	0.09-0.75	=.002
GAD-7^h^	0.50^b^	0.19-0.77	=.004	0.52^b^	0.20-0.83	=.003

^a^PedsQL Gastro: Pediatric Quality of Life Inventory Gastrointestinal Symptom Scale.

^b^Significant effect sizes.

^c^PedsQL QOL: Pediatric Quality of Life Inventory.

^d^CSI-24: Children’s Somatization Inventory-24.

^e^CSI-24 (gastro): Children’s Somatization Inventory (gastro).

^f^ARCS: Adult Responses to Children’s Symptoms.

^g^PHQ-9: Patient Health Questionnaire-9.

^h^GAD-7: Generalized Anxiety Disorder-7.

#### Medication for Abdominal Symptoms

At pretreatment, 12 children were on medications for their abdominal symptoms. At posttreatment, 6 of the children had stopped taking medications, and 6 children were still taking them. None initiated new medications during treatment.

#### Adverse Events

Parents reported that 7 children had experienced an adverse event during the treatment. These events were sleep problems (n=2), increased problems with defecation when decreasing medication for constipation (n=1), lack of time for school homework and other obligations (n=1), longer toilet visits (n=1), increasing number of conflicts due to the treatment exercises (n=1), and feelings of panic once when doing a difficult exposure exercise (n=1). One event was rated as having a big negative impact at the time (sleep problems), two as having medium negative impact at the time (longer toilet visits and increasing number of conflicts and resistance to do the exercises), two as having a small negative impact at the time (sleep problems and difficulty with decreasing medication for constipation), and two were rated as having no impact at the time of the occurrence (lack of time for school homework and other obligations and feelings of panic once when doing a difficult exposure exercise). At the posttreatment assessments, three parents rated that their child was still affected by the adverse events, one with a medium negative impact (sleep problems) and two with a small negative impact (sleep problems and longer toilet visits).

## Discussion

### Main Results

To the best of our knowledge, this is the first study of exposure-based Internet-CBT for children aged 8-12 years with P-FGIDs. The results showed that children and their parents perceived exposure-based Internet-CBT as a feasible, acceptable, and helpful intervention. The within-group effect size was large on the primary outcome measure of gastrointestinal symptoms, from pretreatment to posttreatment, and almost all secondary measures showed significant improvements. Results were maintained or further improved at 6-month follow-up. These results add to the support for exposure-based Internet-CBT for adults and adolescents with IBS [17,19,59,60]. Comparison with other studies in the field is complicated by differences in research designs and by different ways of reporting results: effect sizes are not frequently reported and even mean raw scores are reported differently across studies. In the largest study of CBT for pediatric P-FGIDs conducted by Levy [22], Cohen *d* effect sizes were reported only at 12-month follow-up [61]. The within-group effect sizes in Levy’s study were comparable with our results. Even though the intervention studied by Levy was brief, therapist time per family in treatment was similar between Levy’s and our intervention. In a newly published study of hypnotherapy for children with P-FGIDs [62], the within-group reductions of pain intensity and frequency were large. However, that study reported more modest results on anxiety, depression, and quality of life. We observed larger improvements on these outcomes in this study, especially quality of life. Thus, with the important limitation in mind that the present study did not include a control group and thus causal inferences cannot be drawn, the observed within-group effects are at par with previous studies in the field, indicating potential efficacy of the treatment format and content.

### Strengths and Limitations

Among the strengths of the study were low attrition (with only one child’s assessments missing at posttreatment and three at 6-month follow-up) and high compliance to the treatment. Another strength was the consistent results on the wide range of outcome domains assessed, including abdominal symptoms, quality of life, pain intensity, pain-free days, depression, anxiety, gastrointestinal-specific anxiety, avoidant behaviors, catastrophizing, somatization, sleep, stress, and school absence, and for parents also work absence, responses to the child’s symptoms, parental depression, and parental anxiety. The outcome domains used in the study reflect the extent of problems associated with P-FGIDs and are based on the recommendations for assessments in clinical trials for pediatric recurrent pain [36]. The external validity of this study is strengthened by the fact that participants were recruited via primary, secondary, and tertiary health care for children, and few exclusion criteria were used. In this study, psychiatric comorbidity was assessed with a structured interview (MINI-KID) [31,32] conducted by psychologists. It would have been interesting to compare the psychiatric comorbidity in this study with other studies in the field. Unfortunately, we have found only one other treatment study for children with P-FGIDs where the psychiatric comorbidity was assessed and presented [46]. In that study, the psychiatric comorbidity was comparable with what was observed in our study. Hopefully, psychiatric comorbidity will be assessed and presented thoroughly in future studies to enable comparison and discussion.

The most important limitation of the study is the within-group study design. This design was chosen to match the aims of the study: to assess the acceptability, feasibility, and preliminary within-group effect sizes, before conducting an RCT.

### Possible Mechanisms and Clinical Implications

CBT for children with P-FGIDs typically include multiple components, and thus, several possible mechanisms of treatment [22,46,63]. In this study, we had a distinct focus on exposure to abdominal symptoms and associated stimuli. Fear and avoidance of these stimuli have been established as key maintaining factors in adult IBS [64], and this is likely an important mechanism also for children with P-FGIDs. We observed large effect sizes on the process variables related to decreased avoidance (IBS-BRQ), decreased gastrointestinal-specific anxiety (VSI), and decreased parental protectiveness and monitoring (ARCS protect and ARCS monitor). These results support a model where interoceptive and in-vivo exposure exercises and changed parental responses to children’s symptoms lead to reduced fear and avoidance and thereby symptom improvements. Future studies should perform mediation analyses on these variables to explore how they interplay and affect symptoms.

Pediatric P-FGIDs have been associated with societal costs, such as extensive health care visits [5], school absence, and parental time off work [4]. This study shows that school absence and parental work absence can be affected by the treatment, but because of the study design (with no control condition), no comprehensive health economic evaluation was conducted. Future studies should thoroughly assess and take into account economic factors, when designing and conducting clinical trials for this population, to investigate if there are societal benefits as well as benefits for the families taking part of the treatment [36].

Considering the large effect sizes on the primary outcome measure, the high level of acceptability as rated by both children and parents, and the limited amount of therapist time required, this treatment is highly promising in reducing symptoms, improving quality of life, and increasing accessibility to psychological treatments for children with P-FGIDs.

### Conclusions

This is the first study where children aged 8-12 years with P-FGIDs were treated with therapist-guided exposure-based Internet-CBT. The children and their parents perceived the treatment as acceptable, feasible, and helpful. Despite the long duration of abdominal pain before start of the intervention, improvements were statistically significant on almost all measures from pretreatment to posttreatment, and at 6-month follow-up, all measures showed significant improvements from pretreatment. The within-group effect size on the primary outcome measure PedsQL Gastro was large from pretreatment to posttreatment, and the results were maintained at 6-month follow-up. We conclude that this treatment may be highly feasible and clinically effective. The results need to be confirmed in an RCT.

## Abbreviations

ARCS: Adult Responses to Children’s Symptoms
CBT: cognitive behavioral therapy
CDI-S: Child Depression Inventory-Short version
CSI-24: Children’s Somatization Inventory-24
FAP: functional abdominal pain
FD: functional dyspepsia
GAD-7: Generalized Anxiety Disorder-7
IBS: irritable bowel syndrome
IBS-BRQ: Irritable Bowel Syndrome-Behavioral Responses Questionnaire
Internet-CBT: Internet-delivered cognitive behavior therapy
ISI-C: Insomnia Severity Index-Child version.
MINI-KID: Mini-International Neuropsychiatric Interview for Children and Adolescents
PAS: Pressure Activation Stress Scale
PedIMMPACT: the Pediatric Initiative on Methods, Measurement, and Pain Assessment in Clinical Trials
PedsQL Gastro: Pediatric Quality of Life Inventory Gastrointestinal Symptom Scale
PedsQL QOL: Pediatric Quality of Life Inventory
P-FGID: pain-related functional gastrointestinal disorder
PHQ-9: Patient Health Questionnaire-9
RCT: randomized controlled trial
SCAS-S: Spence Children Anxiety Scale-Short version.
SOL: stop, observe, and let go
VSI: Visceral Sensitivity Index
